# Determining the information needs of contact lens wearers for better education and more support: a qualitative study

**DOI:** 10.1186/s12886-021-02085-0

**Published:** 2021-09-07

**Authors:** Fatemeh Falahati-Marvast, Fateme Alipour, Jamileh Farokhzadian, Leila Ahmadian

**Affiliations:** 1 Department of Health Information Sciences, Faculty of Management and Medical Information Sciences, Kerman University of Medical Sciences, Kerman, Iran; 2 Eye Research Center, Farabi Eye Hospital, Tehran University of Medical Sciences, Tehran, Iran; 3 Nursing Research Center, Kerman University of Medical Sciences, Kerman, Iran; 4 Department of Community Health Nursing, Razi Faculty of Nursing and Midwifery, Kerman University of Medical Sciences, Kerman, Iran

**Keywords:** Contact lenses, Contact Lens wearers, Educational needs assessment, Information needs, Qualitative research

## Abstract

**Background:**

Designing educational interventions tailored to the needs of Contact Lens Wearers (CLWs) are important and necessary. The present study aimed to determine the information needs of CLWs to educate and provide information to them to increase their knowledge and reduce complications and non-compliance behaviors.

**Methods:**

A qualitative approach was applied and semi-structured interviews were conducted in three contact lenses (CL) clinics in Iran among all their practitioners and 24 purposively selected patients. Data were analyzed using the Lundman and Graneheim conventional content analysis.

**Results:**

The qualitative analysis revealed knowledge and skill themes as two main categories. The knowledge category includes five subcategories of basic information for the CLWs, acquaintance with the CL, caring for CL, hygiene and vigilance of CL, and challenges of using CL. The skill category consists of two subcategories, including handling/insertion and removal of the CL, and stabilization of learned information. Moreover, 36 sub-subcategories emerged from these seven subcategories that reflected the information needs of CLWs.

**Conclusions:**

A clear understanding of CLWs’ information needs can help to design and develop appropriate educational approaches to overcome training barriers such as physicians’ time constraints and high workload. Moreover, it can help deal with CLWs’ insufficient knowledge and provide the required information simply and practically with the possibility of enough repetition.

## Background

The use of Contact Lenses (CL) has increased tremendously over the years for a wide range of applications, including vision correction, management of corneal disorders, and cosmetics [1–4]. Approximately 150 million people in the world use CL [2, 5, 6] to correct refractive errors such as myopia, hyperopia, and astigmatism as alternative to spectacles; and as a main therapeutic option in cases such as aphakia, keratoconus, irregular cornea, and high anisometropia which, the glasses cannot help or conditions such as ocular surface problems which need specialized lenses for their management [2, 7].

Despite the advantages of using CL [8–11], adverse reactions with different severities may occur from clinically inconsequential to potentially vision-threatening. Discomfort, corneal staining, dryness, microbial keratitis, corneal neovascularization, allergic conjunctivitis, and corneal infection are the main complications limiting the success of the CL and eventually may lead to the discontinuation of CL [5, 7, 10, 12–18]. One study showed that 6% of Contact Lens Wearers (CLWs) have experienced a complication each year [18] and another study in the U. S reported that one-third of the CLWs had a CL-related condition requiring a physician’s visit [19].

Most CL-related complications occur due to noncompliance with recommendations and CL care, including improper cleaning and disinfection, using the CL for more than the recommended period, incorrect use of the solution, and inadequate handwashing [13, 20, 21]. To do proper CL care and maintenance, CLWs need adequate knowledge and awareness to minimize or prevent complications [13, 16, 21]. Acquiring relevant information is an important aspect of care and support for CLWs that enable them to have greater control over their self-care [22, 23]. Therefore, CLWs should be trained – both at the beginning of CL use and periodic throughout CL wearing – to increase their knowledge, modify their behavior, and enhance their motivation about CL care and hygiene [21].

Various studies investigated the level of CLWs compliance and the impact of education on the compliance behavior of wearers and developed strategies to aid them regarding their compliance and success in using CL [20, 24, 25]. Şengör et al. assessed the consumers’ knowledge and perception of the CL to provide tips and recommendations for education and to increase consumers’ awareness [26]. Other studies [10, 16, 17, 21, 22, 27–32] measured the relationship between awareness, knowledge, compliance, and complications of CL. Various studies have demonstrated the need for training and increasing information about the use of CL [22, 24, 29].

A training program should meet individuals’ needs to be effective [33]. The users are valuable resources for providing information regarding their needs [33, 34]. The needs assessment provides valuable information for designing educational interventions [35]. To determine the real needs of the indivituals and explore the gaps in existing educational programs, evaluating their perspectives through a qualitative approach could be helpful.

To our knowledge, there is no reported qualitative study evaluating the information needs of CLWs. Existing studies investigated the extent of non-compliant behavior of CLWs and how to use CL to develop strategies in CLWs’ education in order to gain needed information and acquire an appropriate understanding for safer CL wearing [25, 26]. Therefore, this qualitative study aimed to determine the information needs of CLWs and design interventions in the future to increase their knowledge and awareness regarding the use of CL.

## Methods

### Design, Settings, and Participants

The current study utilized a qualitative approach and conventional content analysis to describe the information needs of CLWs. To gain a better understanding of their information needs, the experiences, problems, and concerns of CLWs were explored. This study was conducted in three CL clinics in Tehran, Iran to achieve maximum variation in sampling. Two of these clinics are affiliated with Farabi and Noor ophthalmology hospitals. The third center was a well-known specialized private ophthalmology clinic prescribing soft CL and its own made Rigid Gas Permeable (RGP) CL. These clinics are the largest and the most referral CL centers in Iran, accepting referred patients from all parts of the country, besides serving to neophytes. The study participants consisted of CLWs, ophthalmologists, and optometrists as practitioners. All practitioners working in the above-mentioned clinics were recruited. CLWs were selected using a random purposive sampling method. Neophyte CLWs, who have received routine clinic training in the same session of the prescription, as well as CLWs, who have used CL for at least 1 week were invited to participate in the study. CLWs, who had not yet been trained, were excluded from the study.

### Interview Guide

In-depth, semi-structured interviews were performed in person at a convenient place in the clinics from July to September 2019, and data collection was continued until data saturation. Main questions were as follows: 1. What information do CLWs need to use the CL?, 2. What training do they want to receive regarding using the CL?, 3. What information and training do they receive during the first visit of lens prescription and what information is required but not provided?, 4. What information is necessary for them after receiving the lens and going home that they don’t have?, 5. Have they ever had a problem or complication in using CL? If yes, how have they fixed it and what information do they need to solve it?, 6. What concerns do they have about CL?, 7. What information do experienced CLWs consider necessary for beginners?. Every interview took between 35 and 70 min, which was recorded by a voice recorder, besides note-taking. Another interview session was scheduled for participants, who needed more time to think about the questions.

### Data Analysis

Content analysis is a subjective interpretation of textual data to obtain new knowledge and insights when research and literature are limited in the context of the intended phenomenon [36].

Graneheim and Lundman’s content analysis approach [37] was employed for the analysis of the interviews. All interviews were recorded and transcribed verbatim by one researcher (FFM). Transcripts were repeatedly read for a comprehensive understanding of their contents and the creation of meaning units. Then, the data were classified using the inductive method. The meaning units were condensed and then similar content was coded with the same codes. Codes were compared based on their similarities and differences and classified as sub-subcategories. The sub-subcategories with similar meaning and concept were sorted into subcategories. Finally, the main categories as a latent meaning of a text were obtained with the continuous comparison of the similarities of the subcategories. Although the analysis process was systematic, there was a back-and-forth movement between the whole and parts of the text to achieve the most internal consistency and least external incompatibility. Two researchers performed all of these processes. All researchers investigated codes, sub-subcategories, subcategories, and main categories and reached a consensus. The MAXQDA 10 software was used for data analysis.

### Trustworthiness of Data

Lincoln and Guba criteria [38] were considered to confirm the accuracy and strength of the study findings, including confirmability, dependability, credibility, and transferability. To achieve confirmability, all details, including how the work was done and how the data were collected, were carefully recorded. Maximum variation in the selection of the participants was implemented. Two experts in qualitative research checked the process of the work and the research findings. Moreover, the research documents including raw data, notes, transcripts, recorded audio of the interviews were stored for possible future review. Dependability of the data was verified by two members of the research team, who encoded the data separately and reached a final agreement through discussion. The transcripts were shared with the participants and their feedback was received. Credibility was ensured through holding frequent debriefing sessions between researchers and long-term engagement between the participants and the researcher. For transferability of data, details regarding the methodology were described accurately to use findings in other contexts and future consideration.

## Results

Thirty-three participants both male and female were interviewed, 24 CLWs and 9 practitioners including ophthalmologists and optometrists (with Bachelor’s and Master’s academic degrees). Four participants asked for another interview.

**Fig. 1:**
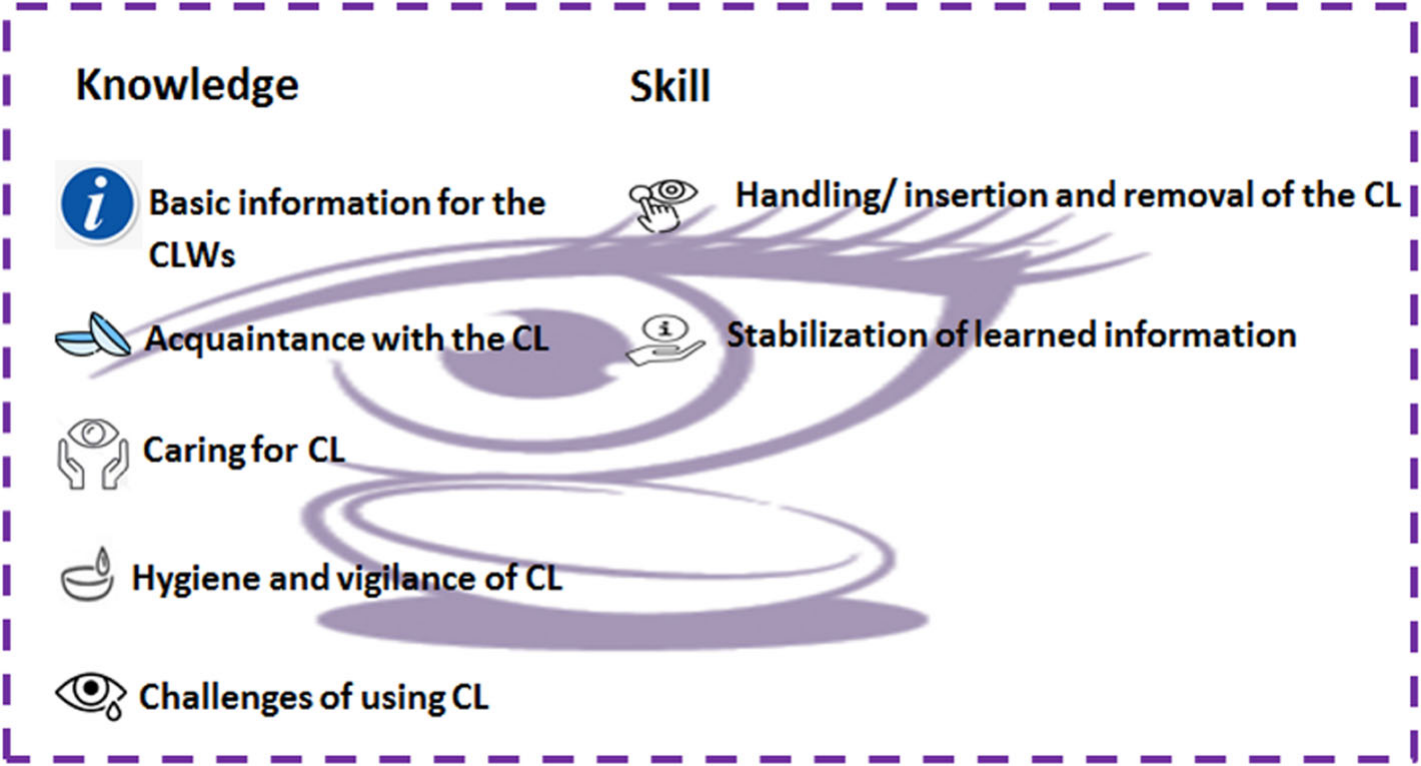
The main categories of knowledge and skill with their seven sub-categories

Table 1 shows the participants’ demographic information. Some needs were identified jointly by CLWs and practitioners, but some of them were described only by either CLWs or practitioners (Tables 2, 3). Two main categories have emerged from the analysis of data, including knowledge and skill with seven subcategories that are displayed in Fig. 1. There were 36 sub-subcategories, which belong to the subcategories of basic information for the CLWs, acquaintance with the CL, caring for CL, hygiene and vigilance of CL, challenges of using CL, handling/insertion and removal of the CL, and stabilization of learned information. The sub-subcategories and examples of interview excerpts related to knowledge and skill themes are shown in Table 2 and Table 3.

**Table 1:** Demographic information of the participants

Contact Lens Wearers						
Participant	Gender	Age	Education	Indication	Lens Type	Contact Lens Wear Duration
**P1**	F	26	Bachelor	KCN	Corneal RGP	> 3 years
**P2**	F	34	Master	Refractive error	Soft	> 3 years
P3*	F	4	Illiterate	Aphakia	Corneal RGP	> 3 years
**P4**	F	16	Student	Irregular cornea	Corneal RGP	1–3 years
P5*	M	8	Student	Eye injury	Corneal RGP	Neophytes
**P6**	F	30	Bachelor	Refractive error	Soft	> 3 years
**P7**	F	28	Bachelor	Irregular cornea	Corneal RGP	1–3 years
**P8**	M	27	Master	KCN	Corneal RGP Scleral RGP	1–3 years Neophytes
**P9**	F	36	Master	KCN	Corneal RGP	> 3 years
**P10**	F	42	Master	KCN	Corneal RGP	> 3 years
**P11**	M	39	Master	KCN	Corneal RGP	> 3 years
**P12**	M	27	Bachelor	KCN	Corneal RGP	Neophytes
**P13**	M	48	Master	KCN	Corneal RGP	> 3 years
**P14**	F	31	Bachelor	Refractive error	Soft	> 3 years
**P15**	M	51	Master	KCN	Corneal RGP	> 3 years
**P16**	M	44	Bachelor	KCN	Corneal RGP	> 3 years
P17*	M	3 Months	Illiterate	Aphakia	Corneal RGP	Neophytes
**P18**	M	24	Bachelor	KCN	Corneal RGP	Neophytes
**P19**	M	37	Diploma	KCN	Corneal RGP	Neophytes
P20*	M	5	Illiterate	Eye injury	Corneal RGP	1–3 years
**P21**	M	37	Associate degree	KCN	Corneal RGP Scleral RGP	1–3 years 1–3 years
**P22**	M	22	Diploma	KCN	Corneal RGP	Neophytes
**P23**	M	29	Diploma	KCN	Corneal RGP	1–3 years
**P24**	M	41	Diploma	KCN	Corneal RGP Scleral RGP	Neophytes Neophytes
**Eye Care Practitioners**						
**Participant**		**Profession**		**Work Experience (Year)**		**Work Experience in CL Clinic (Year)**
**P25**		Optometrist		25		20
**P26**		Optometrist		13		11
**P27**		Optometrist		10		9
**P28**		Ophthalmologist		11		10
**P29**		Ophthalmologist		2		1
**P30**		Ophthalmologist		41		37
**P31**		Optometrist		12		10
**P32**		Optometrist		34		30
**P33**		Optometrist		10		8

*The mothers answered the questions *P*Participant ***RGP*** **Rigid Gas Permeable** ***KCN*** **Keratoconus**

**Table 2:** Interview excerpts supporting key factors related to the ‘Knowledge’ category

Sub category	Sub-subcategory	Example of Interview Excerpts
1.Basic information for the CLWs	1.1 Familiarity with eye structure and its function ^*^ 1.2 Necessary eye examinations for CL prescription ^**^ 1.3 Indispensable preparations before going to the CL clinic ^**^ 1.4 Accredited centers for prescribing and selling CL ^***^ 1.5 Common CL brands ^***^ 1.6 Information Resources ^*^	1. “Provide more specialized information to CLWs. Explain about the eye structure and how different lenses are placed on its surface. How the eyes and eyelids interact with the CL and provide better vision.”(P15) 2.“CL fitting is performed with trial lenses and age, lens history, visual acuity, eyelids, eye tears, and eye measurement such as corneal curvature are also examined. Each trial lens is placed in the eye for half an hour and examinations are done to determine whether it is appropriate or not. CLWs should be aware of the examinations before prescribing the lens and know that this process is time-consuming and requires patience.”(P30) 3. “Normally, CL is not given to patients during pregnancy and breastfeeding. If the CLW has had a hard CL, he should stop wearing lenses for two weeks before coming to the clinic; three-four days is enough in case of using soft lenses.”(P27) 4. “People should search for authorized centers and qualified individuals, who prescribe lens, and support and save CLWs quickly. The pharmacy or beauty salon is not qualified to prescribe and sell CL.”(P30) 5. “My friends ask me about the brand of my lenses. Some of my friends used invalid brands and had red eyes. I always recommend them buying a good brand.”(P14) 6. “The practitioner gave me just a brochure explaining about lenses and hygiene after prescribing the lens. There was no source to read and ask about the lens. I wanted much more information.”(P2)
2. Acquaintance with the CL	2.1 CL introduction and types of them ^***^ 2.2 CL applications ^***^ 2.3 CL advantages and disadvantages ^*^ 2.4 Reasons to wear CL ^***^ 2.5 CL lifespan ^***^	1. “I want to know about the structure of CL and their types, the difference between soft, hard and oxygen-permeable CL” (P8) 2. “Explain the applications of CL to CLWs. One person has poor eyesight and needs a soft lens to improve vision and another person wants to use it for beauty. Athletes must wear CL for better vision. People with corneal disease use hard lenses such as scleral and RGP.”(P26) 3. “I have keratoconus and used a hard lens (RGP) for short time. It was unbearable. I want to get a scleral lens because I heard it is comfortable. I have to use the lens for a long time during the day and want to know which lens has more advantages and is better for me.” (P8) 4. “It’s very good to give the user some information while prescribing the lens. Explain the individual visibility and extent of the illness and why he/she should use a CL” (P10) 5. “CLWs should know that the lifespan of lenses varies depending on the type, and some people use a three-month lens for up to six months. The appearance of the lens may not have changed, but the lens does not have any water and oxygen.”(P33)
3. Caring for CL	3.1 Lens protection ^**^ 3.2 Gradual use of hard CL^*^ 3.3 Follow up examination schedule ^***^ 3.4 CL and climatic conditions ^***^ 3.5 CL at work environment ^*^ 3.6 Emergency and non-emergency CL referrals ^***^ 3.7 Lens care in special groups ^*^ 3.8 When to use and not to use CL ^***^	1. “We explain to CLWs that they need sunglasses for protection CL from wind, dust and sunshine. They must not use sprays or powder shadows and expose to hot places or hair dryers and smoking.” (P33) 2. “During the first days, I was unaware and wore CL from morning to evening that caused me to get headaches. I later found out that I had to use the lens gradually, for example, one hour on the first day, two hours on the second day.”(P4) 3. “I have keratoconus and refer to my doctor according to the time appointed by her to change my lens if necessary. The examination is necessary and the time a doctor visits the patient is different from one case to another. Sometimes the lens can still be used if the lens is intact.” (P13) 4. “CLWs should know what to do during dust scattering, I had to quickly find a place to go and take my lenses out.” (P14) 5. “I had no problems with my lens in the office, but as soon as I entered the building site, the dust went inside the lens and it was no longer possible to use the lens, so I had to take my lens out.” (P15) 6. “I want to know when to see a doctor and what symptoms are dangerous so that I have to remove the lens and what the important and emergency symptoms are” (P14) 7. “When I inserted lenses for my baby, the lens fell out of his eye and there was a possibility of breaking the lens. Later I realized that my baby should be in a lying position about five minutes after the lens insertion to prevent it from falling out of his eyes. The necessary tips about CL in children should be explained for mothers.” (P17) 8. “CLWs should be aware of the dangers. I had a patient who had not taken his/her lens out of his eye for 1 months, and he will certainly tell other people that there is no need to remove the lens daily. It is very helpful to recognize what happens if the lenses are not taken out for several days.”(P31)
4. Hygiene and vigilance of CL	4.1 Disinfection of CL and CL cases ^***^ 4.2 CL solutions ^***^ 4.3 CL maintenance ^***^ 4.4 Makeup materials and CL ^***^	1. “CLWs do not know how to disinfect CL and CL cases and come back to the clinic with eye redness and infections. They must disinfect the CL and container with a special solution. They should place the CL container in boiling water for five minutes to sterilize and replace it every three months.”(P30) 2. “I need to know where to put my solution and normal saline. Doctors should explain us about the solutions we need, how to use and maintain it, and whether it should be kept in the refrigerator or not” (P5) 3. “I bought another lens to use when my lens expired, I did not know how to maintain it. Also I didn’t know where to keep the lens after opening it, and whether I should keep it in the refrigerator” (P5) 4. “I have been wearing CL for a long time. In my opinion, CLWs should be taught how to wear lenses with makeup and what cosmetic products are the best for the eyes. Women have more problems with wearing CL than men because of the use of makeup.”(P15)
5. Challenges of using CL	5.1 CLWs concerns ^*^ 5.2 CL displacement ^*^ 5.3 CL complications ^***^ 5.4 CL incidents ^*^ 5.5 Intolerance of the lens at the beginning of the use ^***^ 5.6 CL and diseases ^*^	1. “At first, my vision was not good half an hour after putting the lens in my eye. I was worried and thought that my keratoconus was progressing. I stopped wearing CL for two weeks. I went to the CL clinic and expressed my concerns. I was not calm. The doctors explained that my vision would gradually improve. It is better that physicians explain CLWs’ concerns when prescribing CL.” (P8) 2. “My lens was displacing during an eye movement and wind blowing. I was horrified and did not know what to do. I came back home quickly and removed it.” (P23) 3. “The patient’s eye becomes red when using CL. The patient comes to the CL clinic after a long time, who has not used the lens for several months due to redness. He would not take the lens out if he knew about redness. Patients must acquire more information about complications and how to react to them.” (P29) 4. “I need to know what to do if an accident happened. For example, CL torn and broke in my sister’s eye; we did not know how to get the broken lens out of her eye because we did not know where the broken lens had gone.”(P2) 5. “When the patient receives the lens, he should know that the RGP lens takes two weeks to become ordinary and tolerable. After a short time of lens insertion, the patient usually comeback and needs help. We must provide information beforehand.”(P30) 6. “I have migraines and when I use CL, my headache gets worse. At first, I did not know, but later I realized that my lens affected migraines.” (P1)

^*^ Needs described only by CLWs ^**^ Needs described only by Practitioners ^***^ Needs described jointly by CLWs & Practitioners

**Table 3:** Interview excerpts supporting key factors related to the ‘Skill’ category

Sub category	Sub-subcategory	Example of Interview excerpts
6. Handling / insertion and removal of the CL	6.1 Practical learning to insert the CL *** 6.2. Practical learning to remove the CL *** 6.3 Practical learning how to wash the hands *** 6.4 Practical learning how to wash the CL ***	1. “I had trouble putting my lens in, so I stopped using the lens for a while. It took me about 45 min to an hour to put the lens in. I searched YouTube for insertion, but I did not learn.”(P12) 2. “It was difficult for me to remove the lens. My wife helped me remove the lens. I thought that it is very easy to put in and take out the lens, but it was not. It took me a long time to learn.” (P23) 3. “The principles of proper hand washing should be educated. CLWs should learn how to use normal saline after washing with tap water. They have to learn whether to use soap or hand washing liquid, whether hand washing is sufficient or disinfection is also needed.”(P22) 4. “Fat and protein settle on the surface of the lens, so it becomes dirty over time. Dirty lenses can damage the eyes. Patients should learn to wash their lenses with a special solution and normal saline after each use according to explanations. Every once in a while, they have to clean the lens surface with special pads and a diluted bleach solution.”(P30)
7. Stabilization of learned information	7.1 Practice and repeat insertion and removal of the CL after the first learning * 7.2 Repetition of information * 7.3 Learning through video *	1. “After practical training of insertion and removal of the lens in the clinic, when I wanted to repeat this training again at home, I had trouble doing this process. I realized how to do it after putting in and taking out lenses several times.”(P9) 2. “Because of the low time in the clinic, it was better that the information about the lens was repeated for me. I fully concentrated on how to remove the lens and did not understand the rest of the content about hygiene, washing and so on.”(P1) 3. “After taking the lens, I was looking for more tutorials. I read texts about lenses from the Internet, and each of them explained different principles step by step. I learned all the content but did not help me. I was looking for videos. Videos are much better for learning. Videos should be made on various lens topics due to their few numbers.”(P8)

* Needs described only by CLWs ** Needs described only by Practitioners *** Needs described jointly by CLWs & Practitioners

### Main category: Knowledge

1. **Basic information for the CLWs**

CLWs need to know fundamental information about CL, including eye structure, eye examinations, prior preparation for going to CL clinic, CL prescription, and sales centers and CL brands. According to the CLWs, having knowelege about the eye and its parts that are involved with the CL is essential for them. This information helps them use the CL with more motivation and less worrisome. According to the practitioners, CLWs’ awareness of necessary eye examinations for prescription allows them to better collaborate with practitioners. Before going to the clinic for an examination, having information regarding prior preparation can prevent ineffective visits and wasting time of CLWs and practitioners and help proper prescription of the CL. CLWs, especially novice users, are unfamiliar with CL and need to know about the valid centers for prescribing and selling CL as well as reputable lens brands to prevent improperly prescribed and poorly fitted lenses or those made from a material not well-suited to eyes. Moreover, access to comprehensive and reliable educational information resources is one of the needs of CLWs to gain awareness and prevent unpleasant experiences (Table 2).

2. **Acquaintance with the CL**

CLWs, especially beginners wanted information about the CL and their lens type. High level of knowledge removes confusion and ambiguity in using CL. Familiarity with CL applications helps answer many CLWs’ questions such as why do some people have soft CL and some have hard CL? When are CL a good alternative to glasses? and when are CL a suitable treatment solution? Recognizing the advantages and disadvantages of different kinds of CL help consumers to have the opportunity to select a proper CL and decide whether to use it or not.

CLWs expressed that understanding the reason for using lenses is very important because it led to easier acceptance of CL and also effective and continuous use of it. CLWs want to know how long the CL will be usable (durability) and whether daily or monthly wearing hours will affect the overall lifespan of the lens (Table 2).

3. **Caring for CL**

After receiving the lens, CLWs wanted to know what instructions will help them take better care of their lenses. According to the participants, each CLWs should be informed of instructions such as:

- Gradually increase your wearing time in hard CLWs (Corneal and Scleral RGP) to be able to use lenses for most of the day (or the final goal of wearing hours)
- When referring to the CL clinic, depending on the type of lens and the follow-up schedule set by the practitioners for re-examination and awareness of the importance of timely and frequent follow-ups
- Learn when to use and not to use lenses e.g. while sleeping, in swimming pools, and during baths
- Do not use tap water for rewetting and/or cleaning CL, do not use CL with long nails, in front of fires and hot environments, do not share CL with others, and do not use CL beyond its recommended duration.
- Do not use CL in warm, humid, and dusty weather
- Notice about certain situations at the workplace such as chemical fumes and vapors, dust, and rays that can be hazardous to CLWs
- Consider emergency conditions, symptoms and signs, which indicate the need for an emergency visit, and the way they deal with these situations.
- Identify non-emergency conditions and the way to deal with these situations.
- Provide certain groups, such as children, teens, and individuals with diseases like migraines and diabetes with adequate information about the effect of their conditions on using CL and vice versa (Table 2).

4. **Hygiene and vigilance of CL**

CLWs have questions about CL maintenance, including how to keep CL when they are not in the eye, the right conditions and temperature for lens maintenance, and how to store extra CL for later use. According to the practitioners, CLWs’ awareness of proper disinfection of CL and its cases leads to the removal of potentially pathogenic germs and decreases the risk of developing an eye infection. The participants stated that disinfectants solutions are an important part of the lens care process. CLWs needed to know about the types of solutions and their brand names, replacement time of CL and its cases, and the use of normal saline for rinsing. They also mentioned that eye makeup was one of the most common challenges for CLWs, and access to instructions helped them apply cosmetics safely and comfortably (Table 2).

5. **Challenges of using CL**

CLWs had many concerns when receiving CL that prompt response to them and raising their knowledge can reduce their concerns. CLWs needed to be informed of probable incidents and the necessary actions while using the lens. Some of them (those using corneal RGP CLWs) experienced CL displacement during the wind blowing, driving (especially by pulling down the car window), or the eye movement and wanted to acquire knowledge for more control over the CL. Lens tolerance was difficult especially for hard CLWs on the first days and they had a foreign body sensation. Due to the lack of knowledge, everyone thinks that he/she is the only one, who has this problem. The participants reported that although the use of CL can treat or improve some of the eye diseases or even reduce their symptoms as an auxiliary tool, it can exacerbate some diseases or their symptoms such as headaches in migraine patients. According to the practitioners, complications are the most common problems for CLWs, but they can keep their eyes healthy while wearing CL by learning and observing a set of tips (Table 2).

### Main category: Skill

6. **Handling/insertion and removal of the CL**

CLWs believed that they should be taught how to put in and take out CL, how to rinse CL, and wash their hands in a practical way. CLWs will waste a lot of their time if they do not receive practical training regarding the insertion and removal of CL and if they want to learn how to insert and remove the lens through trial and error. The practitioners reported that CLWs might stop using CL due to unsuccessful insertion and removal of the lens. Therefore, if CLWs have never practiced this, they must first experience it in the CL clinic where a practitioner can show CLWs how to do it. Cleaning and rinsing the CL and hands before inserting the CL in the eye are the most important aspects in the use and handling of lenses, which prevent infection by learning and doing it properly (Table 3).

7. **Stabilization of learned information**

After initial learning, the CLWs will become skillful with some effort. CLWs mentioned that they forgot a large part of the information received in the clinic and wanted it to be repeated for them. They preferred to receive information through video. Weakness in vision and difficulty in reading textual information were the reasons for their desire for multimedia like video and audio (Table 3).

## Discussion

The present study explored the experiences and viewpoints of CLWs, ophthalmologists, and optometrists about the information needs of CLWs. The results depicted various needs of CLWs, which are classified into two main categories, including knowledge and skills and seven subcategories.

One of the subcategories was “basic information for CLWs”. Each CLW differs in terms of occupation, environmental conditions, tear film properties, corneal status and diameter, and eye anatomical features such as interpalpebral distance and eyelid shape [26]. CLWs’ awareness of these factors facilitates the prescription of lenses, easier eye examinations, and selection of appropriate CL by practitioners. In line with our study, Fogel and Schweizer also mentioned the importance of acquaintance with reputable centers for prescribing and selling lenses. They investigated the purchase of lenses from the doctor’s office, store, and the Internet and found that those, who purchased CL over the Internet or at stores, did not follow some of the FDA CL recommendations and wearers using them could be more at risk for complications such as eye infection. Non-credible websites, non-evidence-based Internet information about CL, lack of checkup for CL by an eye care practitioner after purchasing a lens at a store or over the Internet show the importance of valid centers for prescribing and purchasing lenses [39, 40]. In agreement with our findings, another study showed that the CL brand was important for CLWs, who wanted to know more about CL brands [41]. Donshik et al. noted about the CLWs’ need for information resources and their role in improving CL compliance behaviors [25].

In the present study, “acquaintance with the CL” along with its sub-subcategories was identified as another perceived need of CLWs. In line with our findings, Unnikrishnan et al. demonstrated that CLWs needed guidance on different lens types [4]. Kumar’s et al. investigated the reason for using the CL among CLWs and found that most of them used lenses for cosmetic reasons. In their study, the participants considered beauty as the most common reason for using a lens. In contrast, Khan et al. showed that the majority of CLWs only knew that CL were used to correct refractive errors and a significant number of the participants were not aware of the important indications of CL. These studies suggested that CLWs should be more informed of the reasons for CL use [42, 43]. Other studies reported that CLWs should be informed of the advantages and disadvantages of wearing CL to overcome barriers before using it [44, 45]. Moreover, Asiri et al. explained that the majority of CLWs did not have proper information about the lifespan of CL types [46].

Regarding “caring for CL” with its sub-subcategories, consistent with our findings, Sengor and Wu reported that CLWs needed information regarding lens care and follow-ups as they had improper lens care and irregular follow-ups [26, 28]. Ibanga emphasized that CLWs should gain knowledge about proper care and pay attention to climatic conditions [47]. Zimmerman et al. explained that most of the CLWs were unaware of the risk of exposing CL or storage case to water and practitioners should better educate them to avoid exposing their lenses to any source of water [48]. Chavan et al. noted that 58% of the CLWs were uninformed of acanthamoeba infection related to use of tap water as a cleaning material for CL [49]. Other studies also noted CLWs’ insufficient knowledge of Keeping nails short [13], not sharing the CL with others [45], over wear syndrome and replacement time of reusable CL, and not using CL beyond the expiry date [13, 49, 50]. Moreover, CLWs in special groups like children, teens, allergic individuals, and those with different underlying diseases needed information based on their own condition. Providing tailored information can help the diabetic patients promote CL care behaviors [51], migraine patients wear the suitable CL to prevent CL-related stimulus that can trigger migraine attacks [52], and increase parents/children’s ability in CL use and its care [53]. Jafari et al. showed that one of the most frequent causes for the ophthalmology emergency referral was the problems related to CL and signified a need for further educational and preventive interventions to increase CLWs’ knowledge [54]. Other studies recommended that CLWs should be informed and re-educated when to use and not to use CL such as swimming, entering a hot tub, or taking shower [13, 55].

Another need for CLWs was “Hygiene and vigilance of CL” along with its sub-subcategories. In agreement with our findings, a study showed that information about make-up such as eyeliner, mascara, face and body lotion was often overlooked by practitioners and should be more explained especially to female CLWs [56]. Other studies [13, 43, 56–58] reported that wearer’s inadequate behaviors such as improper cleaning and disinfection of CL and its cases, poor CL maintenance, lack of lens case replacement, solution misuse, reuse of solutions, improper use of the normal saline solution, and rewetting drops occurred due to lack of knowledge and understanding. Acquiring adequate knowledge could motivate wearers to be more vigilant in this regard.

In relation with “challenges of using CL” with its sub-subcategories and consistent with our results, other studies [13, 26, 59, 60] mentioned a need to utilize an approach to reduce concerns about the slip of the lens behind the eye, loss of CL, and sight-threatening infection. Numbers of studies suggest applying effective strategies to make CLWs aware of complications [16, 42, 46], the possibility of CL displacement [60], lens tolerance [61], and possible accidents such as torn or ripped CL [62]. Providing information and paying attention to eye diseases such as keratoconus, which is treated with a CL, is very important for CLWs [63].

“Handling/insertion and removal of the CL” with its sub-subcategories was another need in CLWs. Coincident with our result, Sengor emphasized the necessity of raising CLWs’ awareness by providing basic theoretical and practical education focusing on using and cleaning of CL with ophthalmologist’s supervision [26]. Donshik also suggested that the most desired way was one-on-one patient education in the clinic [25]. Review studies also explained the need to develop strategies for better hand hygiene in addition to routine education [59, 64].

Regarding “stabilization of learned information”, similar studies noted that CLWs forgot a large amount of information (50%) after leaving the first medical visit. CLWs (and practitioners accordingly) are preoccupied with lens insertion and removal at the first session of CL prescription, with a level of stress and anxiety which decreases the ability to gain the information provided about CL. The information must be repeated in a positive and enthusiastic manner. It is insufficient to provide only verbal and written instructions [25, 29, 59]. Content and format of the training should be combined with media such as video, voice, illustrations, and the Internet resources [13, 65].

In this study, both CLWs and practitioners identified a large number of educational needs. Moreover, CLWs specifically identified needs related to the subcategories of basic information, caring for CL, challenges of using CL, and stabilization of learned information. Practitioners should consider these needs and CLWs should receive sufficient information in this regard. Necessary examinations and prior preparations are the needs identified by practitioners. Providing information in this regard could results in better CLWs’ cooperation, reduce CLWs’ visits and practitioner’s workload, and prevent wasting time of practitioners and CLWs.

The results of this study showed different aspects of using CL that CLWs should be aware of them. To prevent CL complications and overcome the challenges of using CL, information needs of the individuals should be considered and proper educational interventions should be developed. The results of this study provide good insights for healthcare authorities to design future interventions to increase the knowledge and skills of CLWs.

### Limitations

Although the viewpoints of different individuals were collected in this study, it is unrealistic to claim all information needs of all CLWs are determined, because depending on the culture and circumstances of each community, there may be some other information needs that we could not define in this study.

Moreover, the current study was conducted in three large CL clinics with a focus on the different types of CL. These clinics accept patients with a wide range of CL indications from a simple refractive error to more complex indications such as keratoconus, trauma, and aphakia from all over the country. Thus, it is recommended that future researches are concentrated on one type of CL since different types of CL have different precautions and cares when handling it.

In addition, when conducting such a research it is recommended to also study the small clinics, where the practitioner may not be that confident in handling CL or may have limited time and resources to train the patients.

## Conclusions

CLWs need accurate and sufficient information to use and care for their CL. Providing appropriate information requires a proper understanding of their needs. A wider understanding of the needs of CLWs will benefit CLWs and eye care practitioners and prevent many complications and factors threatening eye health in CLWs. The findings also indicated the importance of how to convey information to the CLWs. Verbal and written education when prescribing lenses are not sufficient strategies to meet the information needs of the individuals. Therefore, new approaches must be provided to present information in a simple, detailed, and practical way with enough repetition.

## Data Availability

The datasets used and/or analyzed during the current study are available from the corresponding author on reasonable request.

## Abbreviations

CLWs: Contact lens wearers
CL: Contact lenses
FDA: Food & drug administration
RGP: Rigid gas permeable
KCN: Keratoconus
